# Miro, MCU, and calcium: bridging our understanding of mitochondrial movement in axons

**DOI:** 10.3389/fncel.2013.00148

**Published:** 2013-09-10

**Authors:** Robert F. Niescier, Karen T. Chang, Kyung-Tai Min

**Affiliations:** ^1^School of Nano-Bioscience and Chemical Engineering, Ulsan National Institute of Science and TechnologyUlsan, South Korea; ^2^Zilkha Neurogenetic Institute, University of Southern CaliforniaLos Angeles, CA, USA; ^3^Department of Cell and Neurobiology, University of Southern CaliforniaLos Angeles, CA, USA

**Keywords:** mitochondria, axonal transport, MCU, MICU1, miro

## Abstract

Neurons are extremely polarized structures with long axons and dendrites, which require proper distribution of mitochondria and maintenance of mitochondrial dynamics for neuronal functions and survival. Indeed, recent studies show that various neurological disorders are linked to mitochondrial transport in neurons. Mitochondrial anterograde transport is believed to deliver metabolic energy to synaptic terminals where energy demands are high, while mitochondrial retrograde transport is required to repair or remove damaged mitochondria in axons. It has been suggested that Ca^2^^+^ plays a key role in regulating mitochondrial transport by altering the configuration of mitochondrial protein, miro. However, molecular mechanisms that regulate mitochondrial transport in neurons still are not well characterized. In this review, we will discuss the roles of miro in mitochondrial transport and how the recently identified components of the mitochondrial calcium uniporter add to our current model of mitochondrial mobility regulation.

## INTRODUCTION

Mitochondria are vital organelles that provide ATP, maintain Ca^2^^+^ homeostasis, and regulate apoptosis in all eukaryotic cells. For cells with high-energy requirement such as neurons, mitochondria are particularly important for neuronal survival, membrane excitability, calcium buffering, and reliable synaptic transmission (Courchet et al., 2013; Obashi and Okabe, 2013; Shao et al., 2013). In addition, the extremely long processes of neurons pose a unique challenge in distributing mitochondria to the appropriate locations; an inability to maintain the dynamics of mitochondrial transport in neurons can thus cause deleterious effects on their function and physiology (Schon and Przedborski, 2011). It has become clear that dysregulation of mitochondrial transport contributes to pathological changes of neurons, as various neurodegenerative diseases such as Parkinson’s, Alzheimer’s, Huntington’s disease, and Down syndrome are associated with defects in mitochondrial transport or dynamics (Schon and Przedborski, 2011). While these neurodegenerative diseases result from different genetic mutations or arise sporadically, the function of the respective gene products may be involved in common pathways leading to altered mitochondrial transport or dynamics in neurons. However, molecular mechanisms that regulate mitochondrial transport in neurons are not well characterized, and how defective mitochondrial transport leads to neurodegeneration remains unclear.

Mitochondrial transport is known to be mediated by interactions between the mitochondrial adaptor proteins to kinesin and dynein motors, as well as the binding of the motor proteins to the cytoskeleton track (Pilling et al., 2006; Russo et al., 2009; reviewed in Saxton and Hollenbeck, 2012). In spite of this now widely held point of view, it still remains unclear whether internal parameters in mitochondria provide another level of regulation that increases the efficiency of mitochondrial transport in axons. It is generally believed that mitochondrial transport in axons is passive, and the internal state of the mitochondrion is inconsequential. In this view, mitochondrial transport is regulated mainly by extracellular signals that modulate cytoplasmic Ca^2^^+^ influx, which in turn controls mitochondrial transport machinery (Saotome et al., 2008). It was posited that cytoplasmic Ca^2^^+^ level is a key regulator of mitochondrial trafficking in axons and dendrites, and that intracellular Ca^2^^+^ influx impedes mitochondrial movement by affecting the overall interactions between the mitochondrial adaptor, motor, and cytoskeleton track. In contrast, we recently discovered that intra-mitochondrial Ca^2^^+^ plays a critical role in mitochondrial transport in axons (Chang et al., 2011), suggesting that intrinsic signals inside of mitochondria may be actively involved in mitochondrial transport. The intra-mitochondrial Ca^2^^+^ level is mostly regulated by the mitochondrial calcium uniporter (MCU) complex, and three components of the MCU complex have been recently identified: MCU (Baughman et al., 2011; De Stefani et al., 2011), mitochondrial calcium uptake1 (MICU1; Perocchi et al., 2010), and mitochondria calcium uniporter regulator 1 (MCUR1; Mallilankaraman et al., 2012a). In this review, we will discuss the new prospects of miro and the MCU complex in regulating mitochondrial transport in axons.

## Miro: A MULTIFUNCTION PROTEIN INVOLVED IN REGULATION OF MITOCHONDRIAL TRANSPORT

Miro1 is a mitochondrial outer membrane protein in which the N-terminal part contains two GTPase domains separated by two EF-hand domains facing the cytoplasm (Fransson et al., 2003, 2006). It also contains a single transmembrane domain on the C-terminus, with a small three amino acid tail leading into the intermembrane space (Fransson et al., 2006). There are two isoforms of miro, classified as miro1 and miro2, which possess a 60% similarity (Fransson et al., 2003). Miro1 has been shown to play a role in mitochondrial transport in neurons, but a divergent role for miro2, if any exists, has not been explored. Results have shown that overexpression of the two proteins produce slightly different phenotypes on mitochondrial morphology: miro1 produces both aggregated and threadlike mitochondria, while miro2 only generates aggregated mitochondria (Fransson et al., 2006), suggesting that the roles of miro1 and miro2 are slightly different on mitochondrial structure.

**FIGURE 1:**
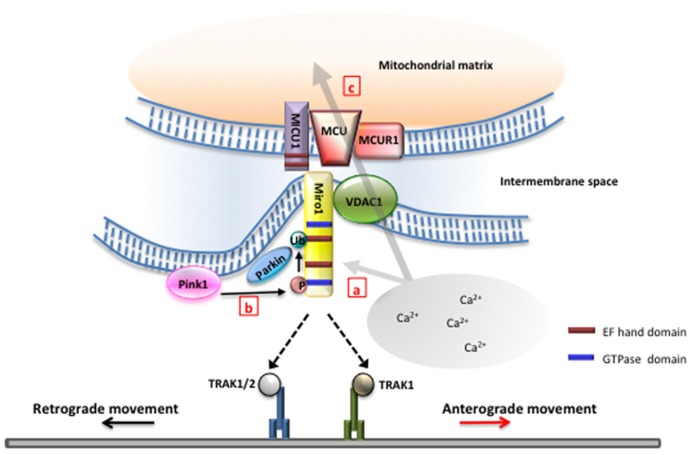
**Miro is the nexus of mitochondrial function and transport.** (a) Miro1 has been proposed to differentially regulate motor–microtubule interaction (Wang and Schwarz, 2009) or TRAK–motor interaction (Macaskill et al., 2009) in the presence of high intracellular Ca^2^^+^. Selective binding of miro1 to TRAK1 or TRAK2 may regulate interaction with kinesin or dynein to modulate directionality of mitochondrial movement (van Spronsen et al., 2013). (b) Phosphorylation of miro1 by PINK1 and subsequent ubiquination of miro by parkin have been found to play a critical role in the initial stages of mitophagy (Wang et al., 2011). (c) Miro has been found to play a role upstream of the mitochondrial calcium uniporter complex in controlling Ca^2^^+^ influx into the matrix. Mutations in the EF-hands of miro1 removes this function (Chang et al., 2011). Ca^2^^+^ influx through the mitochondrial calcium uniporter has also been shown to gate mitochondrial movement (Chang et al., 2011).

Miro1 is known as the primary regulator of anterograde mitochondrial movement along microtubules in axons and dendrites through an indirect interaction with kinesin KIF5B (Tanaka et al., 1998). Normally, miro1 binds to TRAK2 (Brickley and Stephenson, 2011; Milton in *Drosophila*; Glater et al., 2006), a kinesin light chain adapter, which in turn binds directly to kinesin heavy chain and mediates transport along microtubules (Stowers et al., 2002; Glater et al., 2006). However, when cytoplasmic Ca^2^^+^ levels increases, Ca^2^^+^ binding to the EF-hand domains of miro1 results in a conformational shift that subsequently arrests mitochondrial movement (**Figure 1**, a). The mechanism to explain miro1’s role in halting mitochondrial movement is still not clear. Wang and Schwarz (2009) have shown that Ca^2^^+^ binding to miro1 derails the kinesin motor protein from the microtubule track, thereby stopping mitochondrial transport in axons. In contrast, Macaskill et al. (2009) suggested that high levels of cytoplasmic Ca^2^^+^ triggers miro1 to dissociate from kinesin motor together with the adaptor protein, TRAK2, thereby arresting mitochondrial transport.

Studies have suggested that the dynein protein is responsible for retrograde movement. Interestingly, evidences suggest that miro1 is also required for retrograde movement and that kinesin-1 in *Drosophila* must be expressed in order for retrograde movement to occur (Pilling et al., 2006). A recent report demonstrated that miro-associated adapter protein TRAK1 binds to both kinesin and dynein components, while TRAK2 preferentially interacts with dynein (van Spronsen et al., 2013). The differential bindings of TRAK proteins to kinesin and dynein seem to play a critical role in targeting of mitochondria to dendrites and axons, as well as in the bi-directional movement of mitochondria in neurons. However, despite this new information, what regulates the switch between retrograde and anterograde movement still remains unknown.

Recently, miro1 is found to be a substrate of PINK1 (a kinase) and Parkin (an E3 ubiquitin ligase) in neurons (Wang et al., 2011). PINK1 and Parkin are well-known regulators of mitochondrial health and mitophagy (Narendra et al., 2010), and mutations in these proteins are responsible for familial Parkinson’s disease (Kitada et al., 1998; Valente et al., 2001).

Wang et al. (2011) demonstrated that PINK1 phosphorylates miro1, which triggers Parkin-dependent miro degradation, consequently stopping mitochondrial transport in axons (**Figure 1**, b). Overexpression of PINK1 significantly decreases the frequency of mitochondrial movement in axons. Furthermore, PINK1 and Parkin cooperate to remove damaged mitochondria in axons. However, it is to note that mitochondria targeted for degradation did not return to the soma by retrograde movement, but rather remained in axons. While it still remains to be determined if mitophagy indeed occurs in axons, these studies further suggest that miro1 is an important player linking altered mitochondrial movement to mitochondrial damage in axons.

In addition to its role as a regulator of mitochondrial transport, miro1 also interacts with the endoplasmic reticulum (ER). Mitochondria are known to interact through the ER–mitochondria encounter structures (ERMES; Kornmann et al., 2009), which consist of a variety of proteins that tether the two cellular components together. Gem1, a yeast ortholog of miro1, has been found to be one of the proteins responsible for the connection between ER and mitochondria (Kornmann et al., 2011). Gem1, through one of its EF-hands and the two GTPase domains, regulates the interaction between mitochondria and ER. These findings provide additional complexity to the role of miro1 in mitochondrial transport, but raise a possibility that miro1 may play a role in regulating Ca^2^^+^ influx to mitochondria from the ER.

## MITOCHONDRIAL Ca^2+^, Miro, AND MITOCHONDRIAL TRANSPORT

A recent study by Chang et al. (2011) using a genetically encoded green fluorescent protein (GFP)-based fluorescent Ca^2^^+^ indicator targeted to the mitochondria, mito-Case12, suggested mitochondrial Ca^2^^+^ content is another important parameter regulating mitochondrial transport. Chang et al. (2011) discovered that mitochondrial Ca^2^^+^ content correlated inversely with the speed of mitochondrial movement, while no correlation was established for the directionality of mitochondrial transport. Interestingly, this work also offers a new role for miro1 in regulating mitochondrial transport through modulation of mitochondrial Ca^2^^+^ influx. As mentioned above, previous models proposed that increased cytoplasmic Ca^2^^+^ halts mitochondrial movement by binding to miro1 EF-hand domains, thus leading to altered interactions between miro1 and the kinesin motor, or the kinesin motor and the microtubule tracks (Macaskill et al., 2009; Wang and Schwarz, 2009). These conclusions were in part derived from studies using the miro EF-hand mutants which failed to halt mitochondrial transport even in the presence of high cytoplasmic Ca^2^^+^ environment. In contrast, Chang et al. (2011) found that mutations in the EF-hand domain of miro1 reduced Ca^2^^+^ entry into the mitochondria, which is associated with persistent mitochondrial movement. It is thus plausible that blockage of mitochondrial Ca^2^^+^ influx by the miro1 EF-hand mutants also contributes to failure in mitochondrial movement arrest in neurons. These results implicate a potential role for miro1 in regulating mitochondrial movement through regulation of intra-mitochondrial Ca^2^^+^ content, and further imply that intra-mitochondrial Ca^2^^+^ is an important determinant of mitochondrial transport. It is likely that there is a critical Ca^2^^+^ threshold required for pausing mitochondrial movement.

## MCU, MICU1, AND MCUR1: OLD AND NEW PLAYERS IN Ca^2+^ INFLUX TO MITOCHONDRIA

Aside from ATP production, mitochondria are important for Ca^2^^+^ buffering within cells. Ca^2^^+^ influx into mitochondria can stimulate ATP production by stimulating enzymes involved in the tricarboxylic acid (TCA) cycle, the electron transport chain, and ATP synthase complex (Wan et al., 1989). Mitochondrial Ca^2^^+^ buffering allows cells to maintain homeostatic cytoplasmic Ca^2^^+^ level, and under normal conditions Ca^2^^+^ enters the mitochondrial matrix through the MCU complex (Baughman et al., 2011; De Stefani et al., 2011). To further test if Ca^2^^+^ content in mitochondrial matrix regulates mitochondrial transport, Chang et al. (2011) used drugs that inhibit or activate the MCU complex and investigated the mobility of mitochondria. They found that blocking MCU complex in the presence of high cytoplasmic Ca^2^^+^ preserved mitochondrial movement, suggesting that Ca^2^^+^ influx into the mitochondrial matrix plays an obligatory role for mitochondrial movement arrest (**Figure 1**, c). Furthermore, these results identify the MCU complex as a novel component regulating and gating mitochondrial transport. However, mechanisms by which mitochondrial Ca^2^^+^ elevation leads to mitochondrial movement arrest remains to be addressed.

The role and function of the MCU complex were proposed in the 1960s and its properties have been extensively studied, but its molecular nature was only recently identified. Perocchi et al. (2010) first exploited the Mitocarta database of mitochondrial genes in order to search for candidates of the MCU complex *in silico*, then used a targeted RNAi screen for verification. The criteria for the uniporter protein were to have at least one transmembrane domain, a calcium binding motif such as EF-hands, and universal tissue expression. Subsequently, CBARA1, later renamed as MICU1, was identified a candidate protein that met the criteria (Perocchi et al., 2010). Cells having reduced MICU1 by shRNA showed that Ca^2^^+^ influx to mitochondria is prevented even after histamine stimulation that triggers mitochondrial Ca^2^^+^ uptake (Perocchi et al., 2010). Furthermore, MICU1 reduction resulted in an uncoupling of Ca^2^^+^ from mitochondrial energy metabolism without affecting cell survival (Perocchi et al., 2010). However, despite MICU1 having shown many characteristics of MCU, it is still not a channel protein, but a regulator of the uniporter.

Immediately after the identification of MICU1, two independent works discovered the true calcium uniporter that was subsequently named as the MCU. This channel is sensitive to ruthenium red, located in the inner mitochondrial membrane, has calcium channel activity, and interacts with MICU1 (Baughman et al., 2011; De Stefani et al., 2011). MCU contains a novel motif called DIME that consists of Asp-Ile-Met-Glu located between the two transmembrane domains (Baughman et al., 2011; De Stefani et al., 2011). Mutational analysis revealed that these four amino acid residues play critical roles for Ca^2^^+^ influx (Baughman et al., 2011). De Stefani et al. (2011) proposed the MCU topology in which DIME motif faces matrix and two amino termini are present in intermembrane space. On the contrary, other study indicates that the DIME motif is in intermembrane space, while the two protein termini are in matrix (Baughman et al., 2011; De Stefani et al., 2011). This conflicting result should be clarified in future work, but despite the disparity, it is now clear that MCU controls Ca^2^^+^ influx into mitochondria.

Perocchi et al. (2010) showed that histamine-induced calcium influx into the mitochondrial matrix is correlated to the level of MICU1. A recent study, however, failed to reproduce the result (Mallilankaraman et al., 2012b). Instead, Mallilankaraman et al. (2012b) demonstrated that MICU1 regulates MCU-mediated Ca^2^^+^ influx. The authors also identified another regulator of MCU, which is called MCUR1 that binds to MCU and regulates MCU-dependent mitochondrial calcium influx (Mallilankaraman et al., 2012b). It colocalizes with MCU and MICU1 on the inner mitochondrial membrane. Knockdown of MCUR1 causes a decrease in oxidative phosphorylation and calcium uptake, which results in impairment of mitochondrial function (Mallilankaraman et al., 2012a). It is to note that MCU migrates at approximately 450 kDa on native polyacrylamide gel electrophoresis (PAGE) analysis despite of its own molecular weight being about 40 kDa (Baughman et al., 2011). In addition, molecular weight of MICU1 is around 54 kDa (Perocchi et al., 2010) and MCUR1 is 40 kDa (Baughman et al., 2011; De Stefani et al., 2011), respectively. Taken together, this implies that MCU associated with MICU1 and MCUR1 is probably a multi-complex channel with a possibility that more unknown components of MCU machinery have yet to be discovered.

With the MCU complex located in the inner mitochondrial membrane, questions remain as to how Ca^2^^+^ influx into the mitochondrial matrix gates mitochondrial movement. The identification of additional components of the MCU complex will certainly help to delineate the cellular mechanisms. It is plausible that component(s) of the MCU complex is also associated with miro1, and the whole complex modulates Ca^2^^+^ entry into mitochondria as well as mitochondrial transport in axons. Ca^2^^+^ influx through the MCU complex may lead to conformational change in the miro1 protein, subsequently exposing the EF-hand domain for Ca^2^^+^ binding and causing either detachment of mitochondria from the motor or of the motor from the microtubule track. It will be interesting to determine in the future whether miro1 binds to MCU or another component of the uniporter complex.

## CONCLUSION

The MCU complex contains three distinct proteins: MCU, MICU1, and MCUR1, which regulates the influx of calcium into the mitochondrial matrix. It will be particularly interesting to test if manipulation of the identified MCU components can influence mitochondrial transport in the future. In addition, it is possible that miro, which can connect to the ER and regulate mitochondrial movement, would be another component of the MCU complex. Future works involving identification of other MCU components will lead to a better understanding of mechanisms regulating mitochondrial transport in axons.

## Conflict of Interest Statement

The authors declare that the research was conducted in the absence of any commercial or financial relationships that could be construed as a potential conflict of interest.
